# Identification and Validation of an m6A Modification of JAK-STAT Signaling Pathway–Related Prognostic Prediction Model in Gastric Cancer

**DOI:** 10.3389/fgene.2022.891744

**Published:** 2022-07-19

**Authors:** Fei Jiang, Xiaowei Chen, Yan Shen, Xiaobing Shen

**Affiliations:** ^1^ Key Laboratory of Environmental Medical Engineering and Education Ministry, Nanjing Public Health College, Southeast University, Nanjing, China; ^2^ Department of Epidemiology and Health Statistics, School of Public Health, Southeast University, Nanjing, China; ^3^ Department of Occupational and Environmental Health, School of Public Health, Southeast University, Nanjing, China

**Keywords:** gastric cancer, TCGA, prognosis, mRNA, M6A

## Abstract

**Background:** Gastric cancer (GC) is one of the malignant tumors worldwide. Janus (JAK)–signal transduction and activator of transcription (STAT) signaling pathway is involved in cellular biological process and immune function. However, the association between them is still not systematically described. Therefore, in this study, we aimed to identify key genes involved in JAK-STAT signaling pathway and GC, as well as the potential mechanism.

**Methods:** The Cancer Genome Atlas (TCGA) database was the source of RNA-sequencing data of GC patients. Gene Expression Omnibus (GEO) database was used as the validation set. The predictive value of the JAK-STAT signaling pathway-related prognostic prediction model was examined using least absolute shrinkage and selection operator (LASSO); survival, univariate, and multivariate Cox regression analyses; and receiver operating characteristic curve (ROC) analyses to examine the predictive value of the model. Quantitative real-time polymerase chain reaction (qRT-PCR) and chi-square test were used to verify the expression of genes in the model and assess the association between the genes and clinicopathological parameters of GC patients, respectively. Then, Gene Ontology (GO), Kyoto Encyclopedia of Genes and Genomes (KEGG), gene set enrichment analysis, version 3.0 (GSEA), sequence-based RNA adenosine methylation site predictor (SRAMP) online websites, and RNA immunoprecipitation (RIP) experiments were used to predict the model-related potential pathways, m6A modifications, and the association between model genes and m6A.

**Results:** A four-gene prognostic model (GHR, PIM1, IFNA8, and IFNB1) was constructed, namely, riskScore. The Kaplan–Meier curves suggested that patients with high riskScore expression had a poorer prognosis than those with low riskScore expression (*p* = 0.006). Multivariate Cox regression analyses showed that the model could be an independent predictor (*p* < 0.001; HR = 3.342, 95%, CI = 1.834–6.088). The 5-year area under time-dependent ROC curve (AUC) reached 0.655. The training test set verified these results. Further analyses unveiled an enrichment of cancer-related pathways, m6A modifications, and the direct interaction between m6A and the four genes.

**Conclusion:** This four-gene prognostic model could be applied to predict the prognosis of GC patients and might be a promising therapeutic target in GC.

## Introduction

Gastric cancer (GC) is a malignant tumor that occurs worldwide. According to the latest global cancer statistics from 2020, globally, the incidence of GC ranks fifth and the mortality ranks fourth (Sung et al., 2021). At present, the commonly used methods for the treatment of GC include surgery, radiotherapy, and chemotherapy or combination (Song et al., 2017). Although these methods have improved the survival rate of patients, surgery is invasive, radiotherapy is nontargeted, chemotherapy has toxic side effects (Shao et al., 2021a), and the 5-year survival rate of patients is not high (Zhao et al., 2021). Therefore, a prognostic model that can predict the prognosis of GC and provide a new effective target for the treatment of GC should be urgently established.

The Janus (JAK)–signal transduction and activator of transcription (STAT) signaling pathway is involved in gene expression, inflammation, transcriptional programs, and immune response (Meng et al., 2020). Previous research has confirmed that the activation of the JAK-STAT signaling pathway is closely related to many diseases (Yue et al., 2020), including ovarian cancer (Gao et al., 2022), nonsmall cell lung cancer (Prabhu et al., 2021), breast cancer (Chen et al., 2021), and cardiovascular diseases (Baldini et al., 2021).

Some reports regarding the JAK-STAT signaling pathway and GC also exist. Li et al. (2022) found that STAT1 is activated in human *H. pylori*-positive gastritis, whereas STAT1 and its target gene programmed death ligand-1 (PD-L1) are significantly elevated in GC. Bei et al. (2022) found that apatinib enhances GC cell sensitivity to paclitaxel by inhibiting the JAK/STAT3 signaling pathway. Yang et al. (2021) observed that STAM2 knockdown may inhibit malignant processes by targeting the JAK2/STAT3 signaling pathway in GC. These are reports on the association between this pathway and GC, and there are also reports on genes associated with GC that are associated with this pathway. Huang et al. (2022) found that gamma-glutamyltransferase 5 could be a potential prognostic molecular predictor in GC and is involved in the JAK-STAT signaling pathway. Similarly, Lysyl oxidase is also a potential molecular predictor in prognostic GC and also participates in the JAK-STAT signaling pathway (2021).

However, few studies have directly analyzed the effect of genes related to both this pathway and GC on the prognosis of GC and explored the underlying mechanism, and we must learn more about the influential genes and related mechanisms to explore effective therapeutic targets for GC.

Thus, in this study, we aimed to identify and explore the key genes involved in the JAK-STAT signaling pathway and GC based on TCGA and GEO databases. Differentially expressed genes were identified to construct a GC prognosis-related model by following a series of bioinformatic analyses to ensure the predictive value of the model, as well as training test set verification. Moreover, we verified the gene expression and assessed the association between genes and the clinicopathological parameters of GC patients in our samples. Afterward, we explored the mechanism of the genes in the model, which plays a role in the progression of GC via Gene Ontology (GO); Kyoto Encyclopedia of Genes and Genomes (KEGG); gene set enrichment analysis, version 3.0 (GSEA); and sequence-based RNA adenosine methylation site predictor (SRAMP) online websites and RNA immunoprecipitation (RIP)–quantitative real-time polymerase chain reaction (qRT-PCR) experiments. Our results may provide additional evidence about the prognostic biomarkers and therapeutic targets for GC.

## Methods and Materials

### Data Collection

The training RNA-seq data were obtained from the Cancer Genome Atlas Stomach Adenocarcinoma (TCGA STAD) database; the testing RNA-seq data were obtained from GEO (GSE84437). JAK-STAT signaling pathway–related genes were acquired from the GSEA online websites.

### Tissue Samples

A total of 25 pairs of GC tissues and adjacent normal tissues were acquired from GC patients, who were treated in the Department of General Surgery, Nanjing No. 1 City Hospital, from 2015 to 2016 following the Helsinki Declaration. Informed consent was obtained from each patient before they participated in this study. This project was approved by the Ethics Committee of Nanjing Medical University.

### Cell Samples

Gastric cancer cell lines AGS, HGC-27, and gastric epithelial cells GES-1 used in this study were purchased from Saiku Biological Company (Guangzhou, China). All the cells were cultured in Roswell Park Memorial Institute (RPMI) 1640 medium. Both media were supplemented with 10% fetal bovine serum (Gibco, USA) and 1% streptomycin and penicillin (Gibco, USA). Cells were incubated at 37℃ and 5% CO_2_.

### LASSO Cox Regression Analysis and Identification of Different Expression Genes

The glmnet and survival packages were used to construct the LASSO Cox regression analysis. First, the glmnet package was applied to determine the penalty parameter lambda via cross-validation and identify the optimal lambda value that corresponded to the minimum value of the cross-validation error mean. Then, the best gene group was selected to construct a risk model (riskScore model), and the results were categorized into high-risk and low-risk groups based on the median curve. The calculation of the risk score was based on the linear combination of the coefficients obtained from the LASSO Cox regression model multiplied by the expression value of each selected gene. We created a heat map that shows gene expression using the PheatMap software package in R.

Furthermore, the analysis of differentially expressed genes in the GC tissues and adjacent cancer tissues was identified using the limma package. The selection criteria: | log2 fold change| > 1 and *p* < 0.05.

### Survival Analysis, ROC Curve Analysis, and Univariate and Multivariate Cox Regression Analyses

We used the Survival and Survminer packages in R to analyze patient survival and prognosis in the high-risk or low-risk group. The survival curve was plotted using the Kaplan–Meier method, and the log-rank test was used to assess statistical significance.

The survival ROC package was used to perform ROC analysis to analyze the prediction effectiveness of the constructed assessment model. Moreover, the area under the ROC curve was calculated. An area under the curve of more than 0.5 indicated that the model could accurately predict patient survival.

Then, using the survival package in R, we performed univariate and multivariate Cox regression analyses to analyze the independent prognostic role of the riskScore model, which also included age, sex, grade, stage, T stage, M stage, and N stage.

### Quantitative Reverse Transcription Polymerase Reaction

TRIzol reagent was used to extract total RNA from tissues and cells according to the manufacturer’s protocol (GenStar, China). RNA was then reverse-transcribed using a reverse transcription kit (Takara Bio, Japan, RR036A). Quantification of mRNA was performed using an SYBR Green PCR Kit (Yeasen Biotech Co., Ltd., China). GAPDH was used to normalize mRNA levels. The primers were presented in Supplementary Table S1A.

### Functional Annotation, Protein–Protein Interaction, m6A Modification of Genes, and Correlation Between Genes

ClusterProfiler package was performed to visualize and compare multiple GO and KEGG] enrichment results. In addition, protein–protein interaction (PPI) analyses were performed to investigate the potential molecular mechanisms using STRING V11.5.

GSEA was used to explore the signaling pathways related to GHR of the model in GC. GSEA was carried out between datasets with low or high GHR mRNA expression in TCGA. The low expression group was selected as the reference. Gene set permutations were performed 1,000 times for each analysis to identify significantly different pathways. The normalized enrichment score, nominal *p*-value, and false discovery rate q-value indicated the importance of the association between gene sets and pathways.

SRAMP online website (http://www.cuilab.cn/sramp) was used to predict whether the gene contains m6A modifications.

Corrplot package was used to analyze the correlation between m6A regulators and genes of the model.

### RNA Immunoprecipitation Experiment

According to the manufacturer’s instructions, first, AGS cells (approximately 1 × 10^7^) were lysed with RIP lysis buffer (EMD Millipore, Billerica, MA, USA). Then, the cell lysates were incubated with RIP immunoprecipitation buffer containing magnetic beads conjugated with rabbit N6-methyladenosine (m6A) antibody (ABclonal, China) and negative control rabbit immunoglobulin G (IgG) (Millipore, Billerica, MA, USA). Samples were incubated with Proteinase K, and then, immunoprecipitated RNA was isolated. Extracted RNAs were analyzed using qRT-PCR to determine whether GHR, PIM1, IFNA8, and IFNB1 could be pulled down by m6A protein significantly.

### Statistical Analysis

R software and Prism 6 were used to analyze all data. Perl language was used to merge all datasets. The Wilcox test or paired *t*-test was used to assess the difference in mRNA expression between GC tissues and adjacent cancer tissues. The difference in overall survival in the low- or high-risk score group patients was analyzed using Kaplan–Meier and log-rank tests. The correlation between risk scores and patients’ clinicopathological characteristics was examined using the Kruskal–Wallis test. The association between gene expression and clinicopathological parameters was analyzed using the χ^2^ test. Univariate and multivariate analyses were conducted based on a Cox proportional hazard regression model. Statistical significance was set at *p* < 0.05.

## Results

### Identification of Different Jak-STAT Signaling Pathway–Related Expression Genes in TCGA

Through the GSEA online website http://www.gsea-msigdb.org/gsea/msigdb/collections.jsp, we obtained 155 genes on the Jak-STAT signaling pathway (Supplementary Table S1B).

**FIGURE 1:**
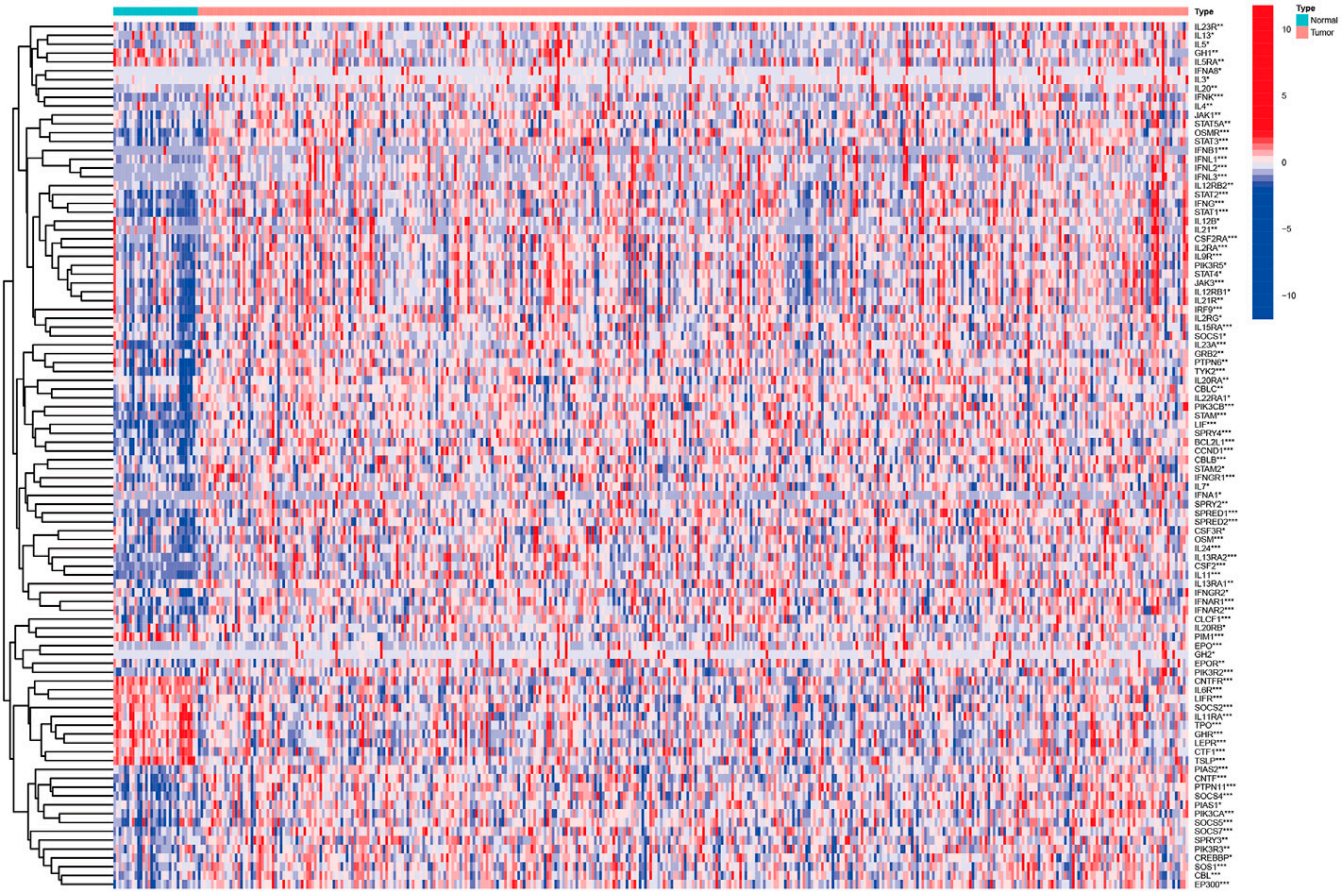
Heatmap identified different expression Jak-STAT pathway‐related mRNAs in GC based on TCGA.

Then, we identified 98 different expression genes with the Wilcoxon test from the STAD dataset in TCGA (The criterion is *p* < 0.05, Figure 1, Supplementary Table S1C).

### Acquisition of the Shared Different JAK-STAT Signaling Pathway–Related Expression Genes via GEO Dataset and TCGA

We obtained a total of 74 species of human results when we used “gastric cancer” and “survival” as the search keywords. In our further analysis of the title and abstract, we selected the GSE84437 data set because of its large sample size (433 samples) and because each sample has the survival index.

When we used *p* < 0.05 and |FC | > 2 as our criterion for screening (according to Supplementary Table S1C, there were 32 genes with *p* < 0.05 and |FC |> 2), 28 DJSEGs were finally obtained, which were shared both in GEO and TCGA (Supplementary Tables S2A,B). At the same time, we used the surrogate variable analysis package for batch correction of data in two datasets. Furthermore, the data used in our subsequent analysis are all normalized data.

### Construction and Validation of the Prognostic Model of DJSEGs

**FIGURE 2:**
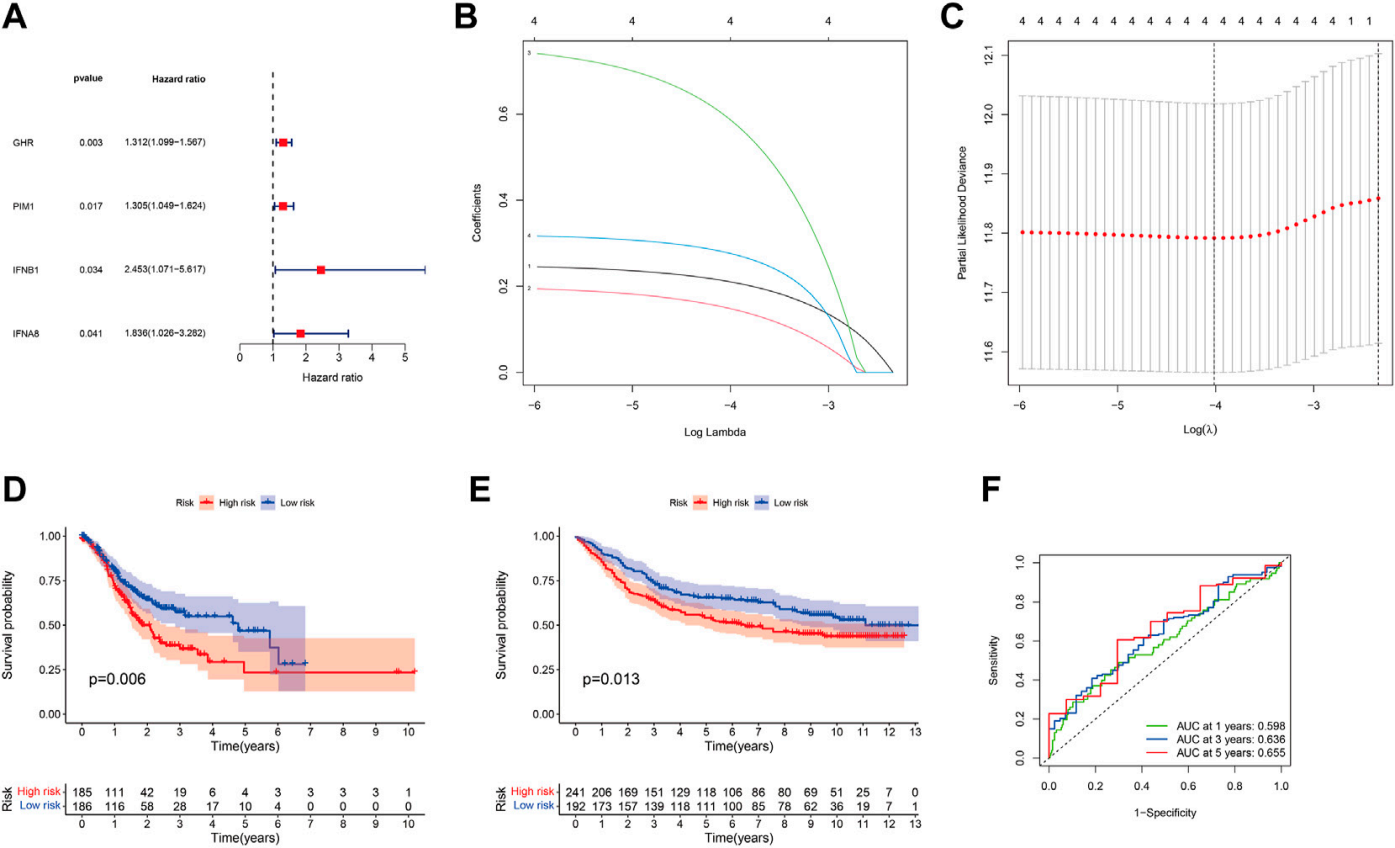
Prognostic ability of the DJSEGs model. **(A)**. Univariate Cox regression analysis identified mRNAs with prognostic values. Hazard ratios were visualized in forest plots. **(B)**. LASSO regression analysis was used to build the final prediction model based on the optimal gene. The number on top of the plot represents the total number of genes. Partial likelihood deviance is plotted against log lambda. Dotted vertical lines were drawn at the optimal values. The optimal gene group was chosen by 10-fold cross-validation and the minimal value of lambda. **(C)**. LASSO coefficient profiles of the four shared genes. The number on top of the plot represents the total number of genes. Each curve represents the corresponding shared gene, and the number next to it is the serial number of each gene; **(D,E)**. Kaplan–Meier survival curves for patients with GC in the training set (TCGA) and the testing set (GEO) stratified by high- and low-risk scores, and high-risk patients had shorter overall survival than low-risk ones. **(F)**. Accuracy of the riskScore model in predicting 1-, 3-, and 5-year overall survival of GC patients, according to the training set (TCGA).

To investigate the effect of shared DJSEGs on GC prognosis, univariate Cox regression analysis was used first. Furthermore, we found that four DJSEGs had prognostic value (Figure 2A; *p* < 0.05). Then, the LASSO Cox regression analysis results suggested that the model worked best when all four DJSEGs were included (Figures 2B,C). The computation of the risk score is elucidated in terms of the expression level of each gene: riskScore = GHR×0.21087289828517 + PIM1×0.149236426661109 + IFNB1×0.589611733452815 + IFNA8×0.277049597016476. The median risk score was applied to categorize patients into high-risk (TCGA: n = 185, GEO: *n* = 241) and low-risk (TCGA: *n* = 186; GEO = 192) groups.

We found that the low-risk group patients had a lower probability of mortality than the high-risk group patients, both in the training set (TCGA normalized data set, Figure 2D) and the testing set (GEO normalized data set, Figure 2E). Thereafter, the area under the ROC curve (AUC) result of the training set was 0.598 at 1 year, 0.636 at 3 years, and 0.655 at 5 years (Figure 2F); the AUC of the testing set was 0.558 at 1 year, 0.565 at 3 years, and 0.572 at 5 years (Supplementary Figure S2A), which indicated that this model could be an indicator for patients’ prognosis.

### The Prognostic Model Was an Independent Prognostic Factor in GC

**FIGURE 3:**
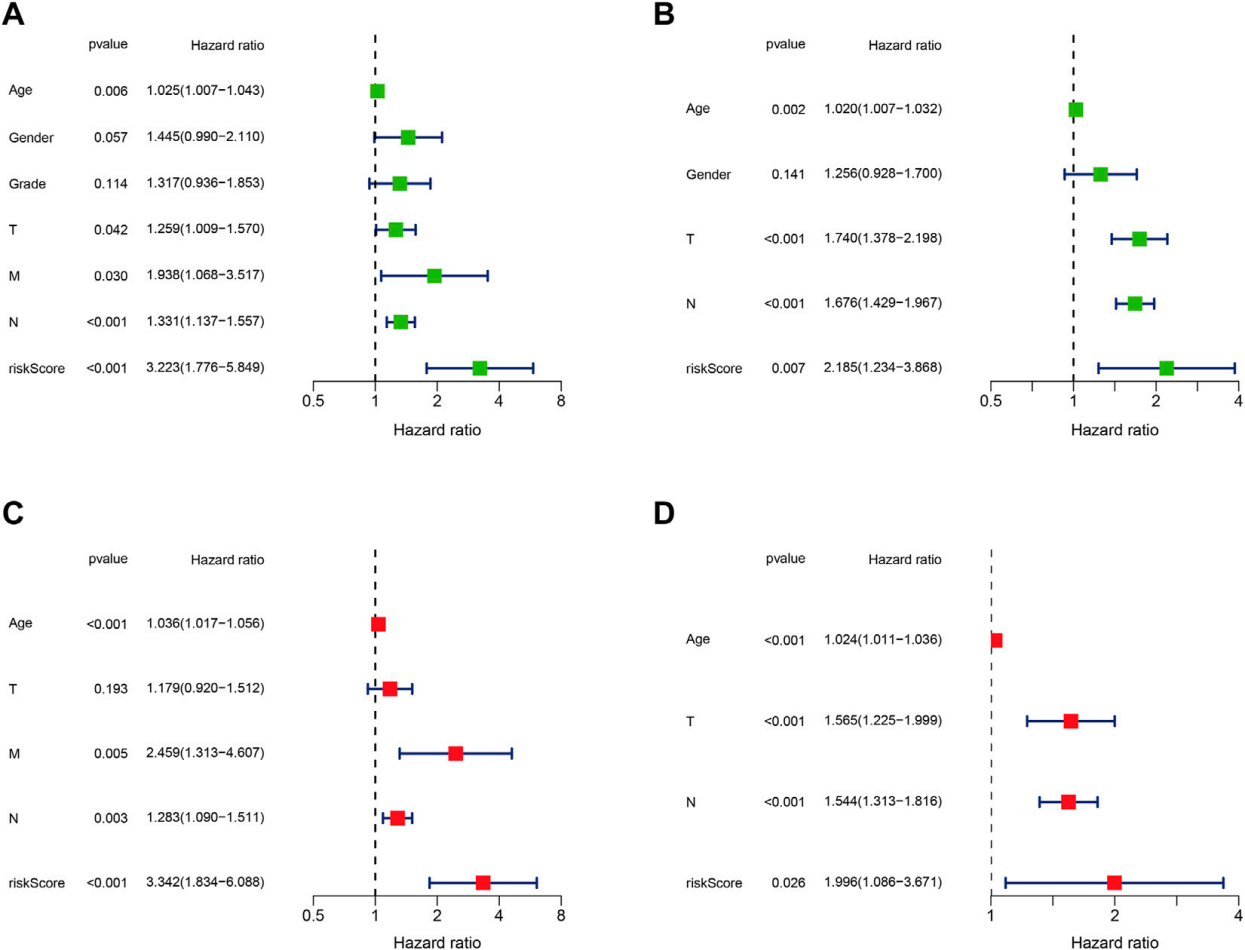
The prognostic model could be an independent prognostic factor in GC. **(A,B)**. Univariate Cox risk ratio analysis of the training set (TCGA) and the testing set (GEO) revealed that the risk model could predict GC prognosis. **(C,D)**. Multivariate Cox risk ratio analysis of the training set (TCGA) and the testing set (GEO) revealed that the risk model could predict GC prognosis independently.

Given the predictive power of the prognostic model, we were interested in determining whether the model could be used as an independent prognostic factor for GC patients. Therefore, univariate and multivariate Cox regression analyses were applied to elucidate the independence of the model. Univariate Cox regression analysis showed that the model of four DJSEGs was significantly related to the overall survival of GC patients. The HR of training set was 3.223 (*p* < 0.001, 95% CI = 1.776–5.849), and the HR of testing set was 2.185 (*p* = 0.007, 95% CI = 1.234–3.868) (Figures 3A,B). Multivariate analyses indicated that this model could be an independent predictor for predicting the prognosis of GC patients (training set: HR = 3.342, 95% CI = 1.834–6.088, *p* < 0.001; testing set: HR = 1.996, 95% CI = 1.086–3.671, *p* = 0.026) (Figures 3C,D).

### The Relationship Between the Expression of Four DJSEGs and Clinicopathologic Characteristics in GC patients

Our bioinformatic results had shown that the four DJSEGs constructed model could predict the prognosis of GC patients, independently. Therefore, we intended to conduct further studies on the expression of these four DJSEGs in our GC cells, patient samples, and their relationship with the clinicopathological data of patients.

**FIGURE 4:**
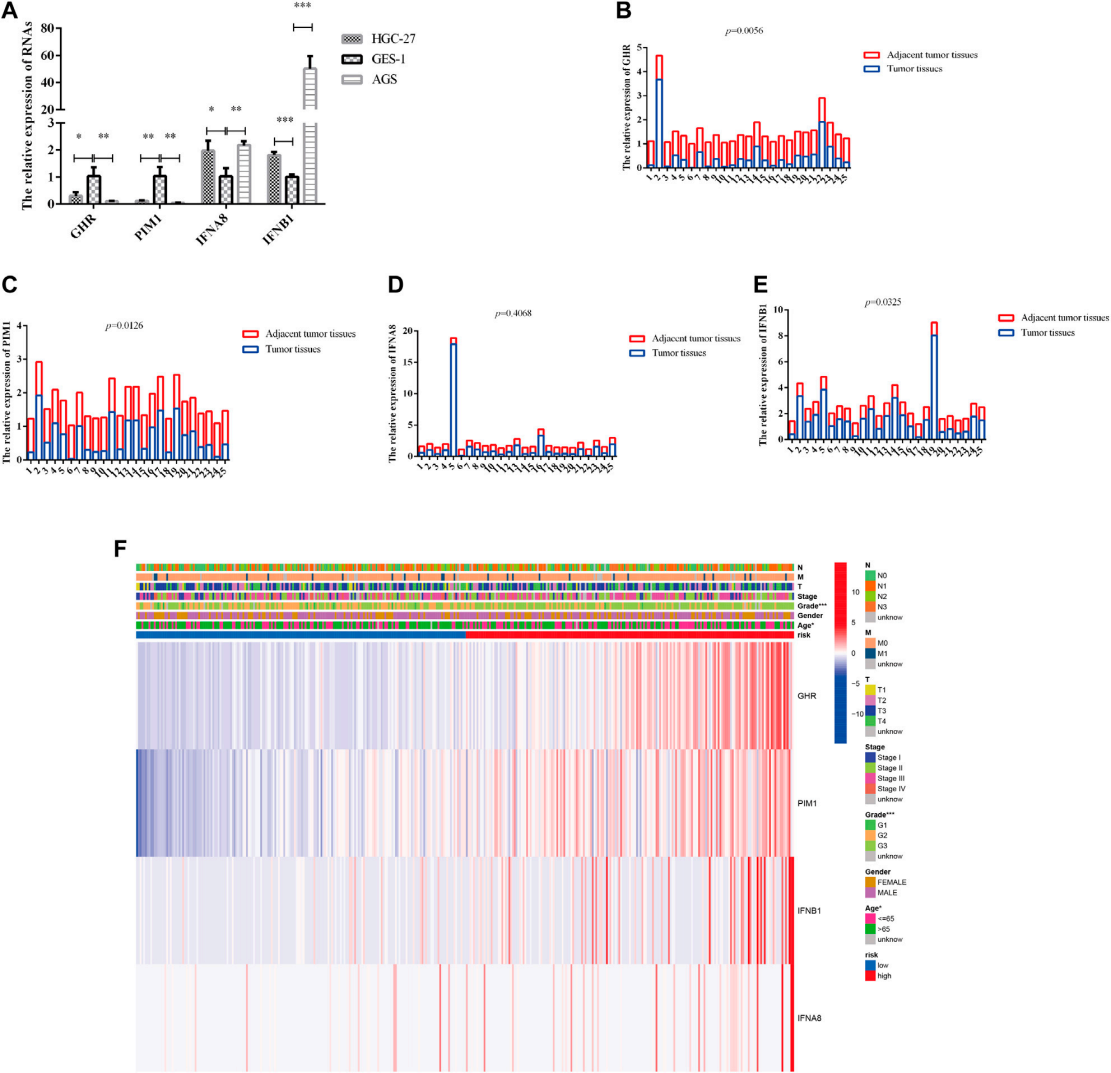
The expression of four DJSEGs in cells, tissues, and the association between four DJSEGs and clinicopathological characteristics in GC patients. **(A)**. The expression of GHR, PIM1, IFNA8, and IFNB1 in HGC-27, AGS, and GES-1. **(B–E)**. The expression of GHR, PIM1, IFNA8, and IFNB1 in 25 pairs of GC tissues and adjacent tumor tissues (the paired sample *t*-test was performed using the 2^−ΔΔCT^ value of each pair of samples). **(F)**. The expression of four DJSEGs and the correlation of clinicopathological parameters with different risk groups are shown in the heatmap. Red indicates overexpression, and green indicates low expression.

According Supplementary Table S1B, GHR and PIM1 were downregulated in TCGA GC patients (GHR: FC = −3.502462468, *p* < 0.001; PIM1: FC = −2.23633752, *p* < 0.001), whereas IFNA8 and IFNB1 were upregulated mRNAs (IFNA8: FC = 20.16667288, *p* = 0.031718147; IFNB1: FC = 3.31414108, *p* < 0.001), in comparison with normal tissues. In our experimental results, we found that the expression trends of GHR and PIM1 were downregulated in HGC-27 and AGS compared with that in GES-1; IFNA8 and IFNB1 were upregulated in HGC-27 and AGS, compared with the expression trend in GES-1 (Figure 4A). Next, we detect all of them in our patients’ sample; the results indicated that the expression trends of GHR, PIM1, and IFNA8 were consistent with TCGA results (Figures 4B,C,E), while there was no difference in the expression trend in IFNA8 between tumor tissues and adjacent tumor tissues (Figure 4D). The results in Table 1 show that only the high- and low-PIM1 groups had different expression trends in different blood types, whereas the rest had no significant statistical differences (In this study, genes were divided into high and low expression groups based on median gene expression levels). Nevertheless, when the model composed of these four genes was analyzed with the clinicopathological data of TCGA patients, it was found that the high- and low-risk groups of the model were significantly correlated with the patient’s age and pathological grade (Figure 4F). These results indicated that four DJSEGs might be involved in the development of GC.

**TABLE 1:** Correlation between the four DIEGs expression and clinicopathological parameters.

Characteristics	Variable	Number(25)	GHR expression		*p*(*x* ^*2*^ *test*)	PIM1 expression		*p*(*x* ^*2*^ *test*)	IFNA8 expression		*p*(*x* ^*2*^ *test*)	IFNB1 expression		*p*(*x* ^*2*^ *test*)
			low	high		low	high		low	high		low	high	
Age (years)	≤65	9	6	3	0.1612	3	6	0.2709	5	4	0.5706	4	5	0.7896
	>65	16	6	10	—	9	7	—	7	9	—	8	8	—
Gender	male	17	7	10	0.3195	7	10	0.3195	8	9	0.8908	8	9	0.8908
	female	8	5	3	—	5	3	—	4	4	—	4	4	—
AJCC (version type 7th)	stage I + II	1 + 13	0 + 5	1 + 8	0.0977	1 + 6	0 + 7	1	1 + 7	6	0.4076	1 + 6	0 + 7	1
	stage III + IV	10 + 0	7	3	—	5	5	—	4	6	—	5	5	—
	NA	1	—	—	—	—	—	—	—	—	—	—	—	—
Tumor size (cm)	≤4.5	13	5	8	0.2191	6	7	0.682	7	6	0.682	7	6	0.682
	>4.5	11	7	4		6	5	—	5	6	—	5	6	—
	NA	1	—	—	—	—	—	—	—	—	—	—	—	—
Blood type	A	5	2	3	0.526	5	0	0.0345	2	3	0.1234	3	2	0.2801
	AB	1	1	0	—	1	0	—	1	0	—	1	0	—
	B	4	1	3	—	1	3	—	0	4	—	3	1	—
	O	15	8	7	—	5	10	—	9	6	—	5	10	—

### The Four DJSEGs Function Analysis Through GO, KEGG, and GSEA

**FIGURE 5:**
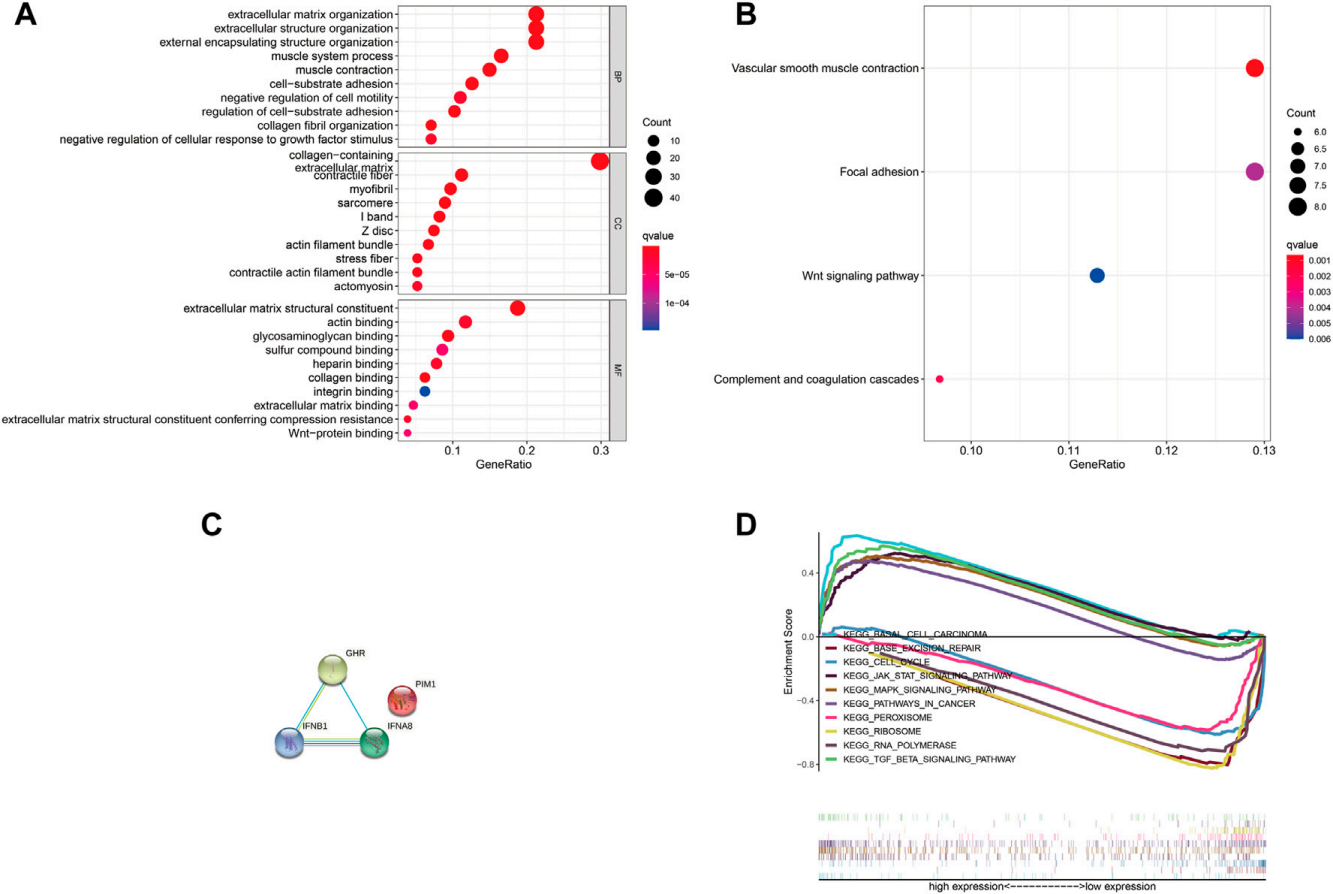
GO, KEGG, and GSEA analysis of the four DJSEGs. **(A,B).** The GO and KEGG network analysis of the four DJSEGs. **(C)**. PPI network of the four DJSEGs. **(D)**. The multiple GSEA analysis of GHR.

Next, we explored the four DJSEGs function by performing GO and KEGG analyses. The GO analysis revealed that the four DJSEGs were mainly enriched in extracellular matrix organization, external encapsulating structure organization, cell-substrate adhesion, and Wnt-protein binding (Figure 5A). The KEGG analysis revealed that the four DJSEGs were mainly enriched in vascular smooth muscle contraction, focal adhesion, and Wnt signaling pathway (Figure 5B). Then, the STRING database was used to explore the interactions of the four DJSEGs, with a confidence score of more than 0.400 (medium confidence). The PPI network showed that GHR, IFNA8, and IFNB1 protein could interact with each other, except for PIM1 protein (Figure 5C). Because the confidence score of GHR interacting with other proteins was higher (Supplementary Table S3A) and the different expression of GHR in our GC patient samples was most significant, we further explored the potential pathways of GHR by regulating the development of GC with the GSEA database. The results showed that “base excision repair,” “RNA polymerase,” “peroxisome,” “ribosome,” and “cell cycle” signaling pathways were enriched in the GHR low expression group; “pathways in cancer,” “basal cell carcinoma,” “mapk signaling pathway,” “TGF beta signaling pathway,” and “Jak-stat signaling pathway” were enriched in the GHR high expression group (Figure 5D). All of these pathways were related to the occurrence and development of GC.

Therefore, it is reasonable to infer that the four DJSEGs model does play a role in the progression of GC and is likely to play the role through these signaling pathways.

### The Four DJSEGs Had Enrichment m6A Modifications

**FIGURE 6:**
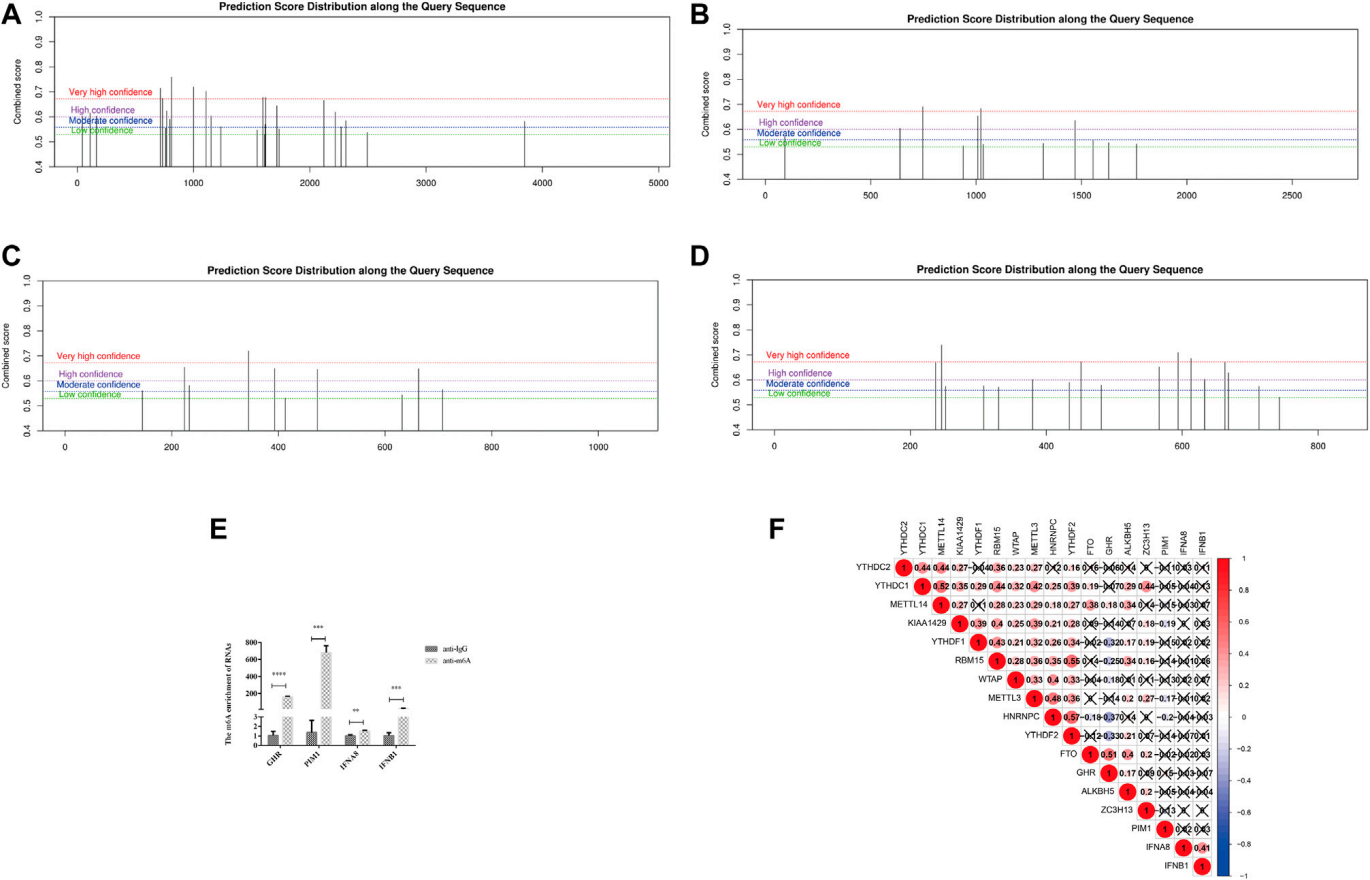
m6A modifications on the four DJSEGs. **(A–D)** M6A modifications of GHR, PIM1, IFNA8, and IFNB1. **(E)**. The RIP-qRT-PCR results of the four DJSEGs. Results indicated that GHR, PIM1, IFNA8, and IFNB1 had enrichment m6A modification than the IgG group. **(F)**. Spearman correlation analysis clarified the association between m6A regulators and four DJSEGs.

In recent years, the m6A modification of noncoding RNA has become a research focus, but m6A modification is more common on mRNA, which plays important regulatory roles in a variety of physiological processes and disease progression (Wu et al., 2021). Therefore, we performed m6A site prediction via the SRAMP online website (http://www.cuilab.cn/sramp), which achieves promising performance both in cross-validation tests on its training dataset and in the rigorous independent tests. The thresholds for very high/high/moderate/low-confidence m6A sites correspond to the thresholds that achieved 99%/95%/90%/85% specificities (in other words, had a 5%/10%/15% false-positive rate) on cross-validation tests, respectively. As shown in Figures 6A–D, very high confidence m6A sites universally existed in the four DJSEGs—GHR, PIM1, IFNA8, and IFNB1.

RIP-qRT-PCR results showed that the m6A antibody could significantly pull down these four genes, indicating that they had direct interaction with the m6A protein (Figure 6E).

To gain further insight into the role of m6A on the four DJSEGs in GC, we studied the correlation between the four DJSEGs (GHR, PIM1, IFNA8, and IFNB1) and m6A writer (KIAA1429, METTL3, METTL14, RBM15, WTAP, and ZC3H13), reader (HNRNPC, YTHDC1, YTHDC2, YTHDF1, and YTHDF2), and eraser proteins (ALKBH5 and FTO) based on TCGA STAD data. The results showed that GHR is strongly positively correlated with FTO and negatively correlated with YTHDF1, YTHDF2, and HNRNPC, whereas PIM1 is weakly negatively correlated with HNRNPC, KIAA429, and METTL3 (Figure 6F). Our RIP experiment results also show that GHR and PIM1 have stronger interaction effects on m6A than IFNA8 and IFNB1.

Thus, we speculated that the function of four DJSEGs on GC progression may also be correlated with m6A modification or m6A regulators.

## Discussion

At present, endoscopic mucosal resection and endoscopic submucosal dissection are the preferred treatments in the early stages of GC (Simić et al., 2019; Min et al., 2021). However, the disease progresses rapidly when patients are diagnosed with GC that are already beyond the early stages; so, the 5-year disease survival rates of GC patients remain low (Li et al., 2018). Chemotherapy regimens, such as SOX (oxaliplatin + S1)/CapeOX (oxaliplatin + capecitabine), FOLFOX (oxaliplatin + leucovorin + 5-fluorouracil), and DCF (docetaxel + cisplatin + 5-fluorouracil)/DOF (docetaxel + oxaliplatin + 5-fluorouracil), are commonly used in patients with advanced GC (Zhang et al., 2020); however, their efficacy is limited. Many current studies suggest the combination of chemotherapy with surgery, radiotherapy, or targeted therapy as a first-line treatment strategy to improve patient survival (Digklia, 2016; Ruan et al., 2020; Mocan, 2021). However, because of the toxicity of chemotherapeutic drugs, the difficulty in screening beneficiaries of targeted therapy drugs, and the tendency of drug resistance, the prognosis of GC patients has not significantly improved (Li et al., 2021; Sun et al., 2021).

However, immunotherapy offers new hope for some cancer patients, and a breakthrough has been made (Li et al., 2021; Liang et al., 2021). In recent years, research on immune checkpoint inhibitors, such as programmed cell death protein-1/PD-L1 inhibitors and anti-cytotoxic T lymphocyte-associated antigen 4 (CTLA-4) inhibitors, has become important for identifying key roles in tumor-induced immunosuppression (Van Limbergen et al., 2017). Nevertheless, some studies have pointed out that not all patients can benefit from immunotherapy. Some researchers have pointed out that tumor mutation burden (Guo et al., 2021), microsatellite instability, and Epstein–Barr virus positivity (Muti et al., 2021) are all correlated with the extent to which patients benefit from immunotherapy, which means that patients need an effective marker to assess their response to immunotherapy. We aimed to identify the key genes related to GC that can be effective independent predictors of immune pathways in GC patients. Thus, we aimed to identify a potential target for the treatment of GC or a biomarker that can reflect the immune response in GC.

In this study, we identified 155 key genes in the JAK-STAT signaling pathway. Thus, we established a refined model that included four different expression genes (GHR, PIM1, IFNA8, and IFNB1). To our knowledge, few studies have reported the function of the JAK-STAT signaling pathway–related key genes in GC. After a PubMed search, we found that only a few pieces of literature reported the role of GHR (Yan et al., 2021; Meng et al., 2022) and PIM1 (Yan et al., 2012; Kim et al., 2020) in GC, whereas there is no relevant report of IFNA8 and IFNB1 in GC at present. In addition, we found that in the four DJSEGs model, the low-risk group patients had a lower probability of mortality than the high-risk group patients, both in training (Figure 2D) and testing (Figure 2E) sets. In the further ROC diagnosis of the constructed model, this study found that the AUC result of the training set was 0.598 at 1 year, 0.636 at 3 years, and 0.655 at 5 years (Figure 2F), whereas the AUC of the testing set was at 0.55–0.58 (Supplementary Figure S2A). In the analysis of related studies on prognostic models, it is found that the AUC of most models is between 0.7 and 0.9, for example, Kunfu Dai et al. found that the AUC of their risk scoring model was 0.75–0.78 for predicting the 1-, 3-, and 5-year overall survival (Dai et al., 2022). In contrast, there are also AUCs below 0.7, for example, Qiansan Zhu et al. found the AUC of the prognosis model was 0.641and 0.677 in forecasting the 2- and 3-year prognosis of rectal cancer, respectively (Zhu et al., 2022). It is generally thought that AUC 0.5 = noninformative; AUC 0.5–0.7 = less accurate; AUC 0.7–0.9 = moderate accuracy; AUC 0.1–1 = high accuracy; and AUC 1 = perfect test (Park and Cho, 2022). Therefore, the model we constructed this time has the predictive ability, but the accuracy is not very high.

In addition, this model was significantly correlated with the patient’s age and pathological grade based on TCGA data, although no statistically significant correlation was found in the clinicopathological parameters of GC patients in our laboratory. When we further analyzed GRHR-related pathways, we found that GHR was highly expressed in a large number of cancer-related pathways and related to some m6A regulators, as well as PIM1. We also found that the m6A antibody could directly interact with GHR, PIM1, IFNB1and IFNA8, suggesting that the function mechanism of four DJSEGs may be related to m6A modifications or m6A regulators.

In recent years, circRNAs have been continuously studied as targets for cancer diagnosis or treatment (Zhang et al., 2017; Arnaiz et al., 2019; Zhou et al., 2019a; Wang et al., 2021). Some researchers have revealed that circRNAs can regulate the expression or function of their parental genes; for example, circEIF3J and circPAIP2 can regulate their parental gene transcription by binding to U1snRNP and RNA Pol II (Li et al., 2015). Other studies suggested that circRNAs could also regulate parental gene expression by acting as miRNA sponges (Zhou et al., 2019b; Kong et al., 2019) and mRNA traps or through translational modulation and posttranslational modification processes (Shao et al., 2021b). In addition, we should consider the influence of miRNA, because miRNA usually regulates the expression or function of its target genes (Zhou et al., 2013; Ho et al., 2017). Such that GHR was regulated by miR-139 (Cui et al., 2017), miR-33a decreased PIM1 expression to inhibit GC cell proliferation (Wang et al., 2015). Therefore, we speculate that the mechanism of the four DJSEGs model influences GC, which may be regulated by upstream circRNAs or miRNAs; however, we still need to verify this through experiments, such as the double luciferase experiment.

According to the above results, the four DJSEGs model could be used as a prognostic indicator of GC patients. Furthermore, they could co-function or be affected by their related pathway genes or proteins, upstream circRNA, miRNA, or their own m6A modification. This study has carried out a comparatively comprehensive prediction and analysis of these four aspects. Thus, this study could be used as a reference basis for future research. In addition, we have to consider the influence of the tumor microenvironment because chronic inflammation and immune cell damage in the tumor microenvironment are also key factors in the development and progression of GC (Zhao et al., 2021).

Nevertheless, this paper has its own limitations. The biggest problem is that the above prediction analysis results lack sufficient experimental verification. We only verified the expression levels of the four genes in GC cells, patient tissues, and their association with m6A, which undoubtedly affected the certainty of the research conclusions.

## Conclusion

Through predictive analysis, we found that GHR, PIM1, IFNA8, and IFNB1 could effectively predict the prognosis of GC, and this predictive ability may be related to their m6A modifications. Overall, our study provides a basis for relevant experimental transformation.

## Data Availability

The datasets presented in this study can be found in online repositories. The names of the repository/repositories and accession number(s) can be found in the article/Supplementary Material.
