# Supplementary figures and images for: Identification and Validation of an m6A Modification of JAK-STAT Signaling Pathway–Related Prognostic Prediction Model in Gastric Cancer
Source: Front Genet. 2022 Jul 19;13:891744. doi: 10.3389/fgene.2022.891744 (PMC9343854; doi:10.3389/fgene.2022.891744)

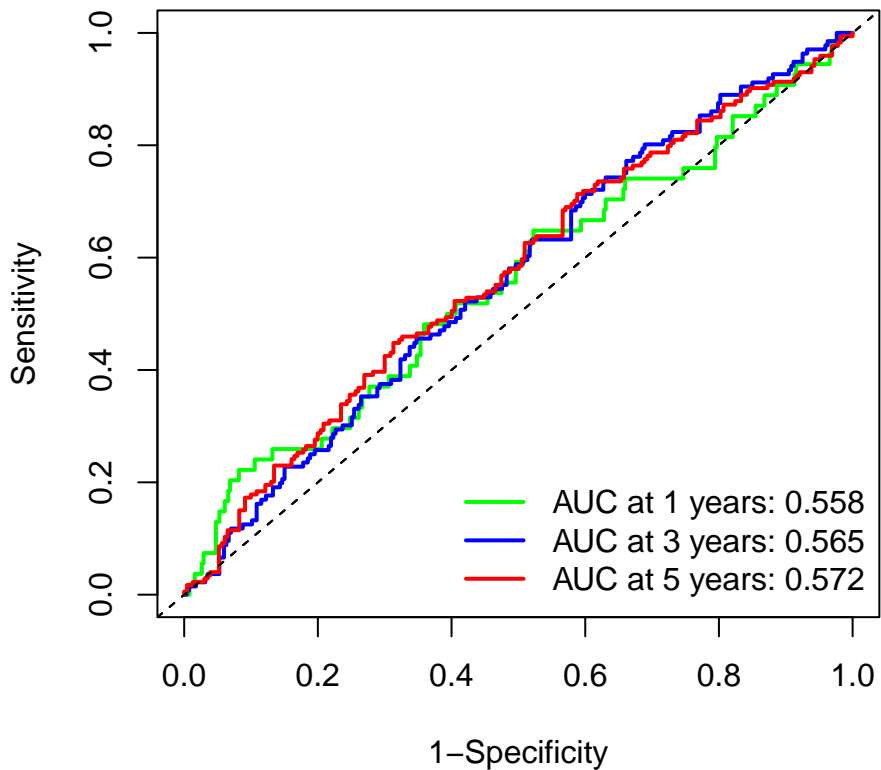

Supplement: Supplementary file 7 [file DataSheet1.PDF]
